# *‘Henicorhynchus’thaitui*, a new species of cavefish from Central Vietnam (Teleostei, Cyprinidae)

**DOI:** 10.3897/zookeys.965.52751

**Published:** 2020-09-03

**Authors:** Dinh Tao Nguyen, Anh Tuan Ho, Ngoc Thao Hoang, Hua Wu, E Zhang

**Affiliations:** 1 School of Life Science, Central China Normal University, Wuhan 430079, Hubei Province, China School of Life Science, Central China Normal University, Wuhan 430079, Hubei Province, China Central China Normal University Wuhan China; 2 Institute of Hydrobiology, Chinese Academy of Sciences, No.7 Donghu South Road, Wuhan 430072, Hubei Province, China Chinese Academy of Sciences Wuhan China; 3 Institute of Ecology and Biological Resources, Vietnam Academy of Science and Technology, No. 18 Hoang Quoc Viet, Hanoi City, Vietnam Vietnam Academy of Science and Technology Hanoi Vietnam; 4 Vinh University, 182 Le Duan street, Vinh city, Nghe An Province, Vietnam Vinh University Vinh Vietnam; 5 Hong Duc University, 565 Quang Trung Street, Thanh Hoa City, Vietnam Hong Duc University Thanh Hoa Vietnam

**Keywords:** Cypriniformes, karst system, morphology, Southeast Asia, taxonomy

## Abstract

*‘Henicorhynchus’thaitui***sp. nov.** is described from a subterranean stream in a karst cave in Phong Nha Ke Bang National Park, Quang Binh Province, Central Vietnam. It differs from all congeners in having a pale pink body in life, smaller eyes with diameter less than the maxillary barbel length, and two pairs of barbels, the maxillary barbel being much longer than the rostral barbel.

## Introduction

Vietnam is rich in karst caves, and there are many magnificent caves throughout the country. The Son Dong Cave is the largest in the world (UNESCO 2016). Many caves, including Vietnam’s deepest cave, were discovered in the northern part of the country, such as Lang Son, Cao Bang, Son La, and Ha Giang. Quang Binh in Central Vietnam is the largest and most important karst region of the country. The Ke Bang limestone massif, which crosses into Laos, has the longest river cave of the world in the Hang Khe Ry and the largest cave passage in the newly discovered Hang Son Doong. Many more caves are expected to be discovered in Vietnam (UNESCO 2016).

Cavefishes are adapted to hypogean aquatic ecosystems. During the past decade, many cavefishes were discovered in Vietnam as a result of ongoing cave explorations (Nguyen et al. 2018). Nevertheless, the fish fauna of those caves remains little studied. Mai (1978) described a single specimen of the family Siluridae from a cave in Cuc Phuong National Park, at the foothills of the Annamite Range in northern Vietnam, as *Silurus cucphuongensis* Mai, 1978, now in the genus *Pterocryptis* Peters (Ng and Kottelat 1998; Ng 1999). Kottelat (2004) described a new loach of the family Nemacheilidae (*Schistura spekuli* Kottelat, 2004) from a cave near Tam Duong, Lai Chau Province, Northern Vietnam. Two other new loaches, *Draconectes narinosus* Kottelat, 2012 and *Schistura mobbsi* Kottelat & Leisher, 2012, were described from Northern Vietnam. Most recently, a new species of Cyprinidae (*Speolabeo hokhanhi*Nguyen et al. 2018) was described from Central Vietnam where it is found only in the Phong Nha-Ke Bang National Park drained by the Son River of the Gianh River basin in Quang Binh Province (Nguyen et al. 2018).

The Phong Nha-Ke Bang National Park is located in the middle of the Annamite Range in the Quang Binh Province. The park, with approximately 104 km of subterranean tunnels and rivers, is among the most outstanding limestone karst ecosystems in the world (UNESCO 2016; Ho et al. 2018). To its West, it is adjacent to the Hin Namno Nature Reserve of Laos. So far, three Laotian cavefish species have been described: *Troglocyclocheilus khammouanensis* Kottelat & Bréhier, 1999, *Schistura kaysonei* Vidthayanon & Jaruthanin, 2002, and *Speolabeo musaei* (Kottelat & Steiner, 2011). More cavefish species are expected to exist in limestone karst areas of the middle Annamite Range (Kottelat and Steiner 2011; Kottelat 2017; Nguyen et al. 2018). This is evidenced by the present report of a new species of cavefish provisionally referred to the labeonine cyprinid genus *Henicorhynchus* Smith, from Phong Nha-Ke Bang National Park, Central Vietnam.

*Henicorhynchus* comprises species widespread in freshwater habitats throughout tropical and subtropical regions of Southeast and South Asia. This genus has a complex taxonomic history. Its included species were previously placed under various genera, such as *Cirrhinus* Oken (Roberts, 1997), and *Gymnostomus* Heckel (Kottelat, 2013). In the absence of a stable classification of parts of the Labeonini we here follow Fricke et al.’s (2020) concept of *Henicorhynchus*, where 9 species are included. The genus has four representatives in Vietnam: *H. siamensis* (Sauvage, 1881), *H. lobatus* Smith, 1945, *H. caudimaculatus* (Fowler, 1934) and *H. cryptopogon* (Fowler, 1935) (Nguyen and Ngo 2001; Serov et al. 2006; Rainboth et al. 2012; Tran et al. 2013). The new species described herein is the fifth species of *Henicorhynchus* and the sixth cavefish species recorded from Vietnam.

## Material and methods

All measurements were taken point to point with a pair of dial calipers and data were recorded to the nearest 0.1 mm. Counts and measurements were made on the left side of specimens whenever possible, following the methods of Rainboth (1996) and Kottelat (2001). Predorsal, prepectoral, prepelvic and preanal lengths were measured from the snout tip to the dorsal-, pectoral-, pelvic-, and anal-fin origin, respectively. Vertebrae were counted from radiographs following the method outlined by Roberts (1989). Weberian vertebrae and the urostylar complex were included in the counts of vertebrae. The number of specimens used for a given count is indicated in brackets after the count. Values for the holotype are indicated by an asterisk. Measurements of parts of the head are given as proportions of the head length (HL). The head length and measurements of other parts of the body are given as percentages of the standard length (SL).

Abbreviations in the text include: IHB, Institute of Hydrobiology, Chinese Academy of Sciences in Wuhan City, Hubei Province, China; IEBR, Institute of Ecology and Biological Resources, Vietnam Academy of Science and Technology in Hanoi City, Vietnam; RIA1, Research Institute for Aquaculture No. 1, Bac Ninh Province, Vietnam; RIA2, Research Institute for Aquaculture No. 2, Khanh Hoa Province, Vietnam; VU, Vinh University in Nghe An Province, Vietnam; CTU, Can Tho University in Can Tho City, Vietnam; CAS, California Academy of Sciences; USNM, National Museum of Natural History, Washington, DC, USA.

### ‘Henicorhynchus’ thaitui sp. nov.

Taxon classificationAnimaliaCypriniformesCyprinidae

F9C85D76-B442-5288-849D-EDE19FA04110

http://zoobank.org/0E2614B1-EEBB-4DDE-B883-EAD279245950

**Figure 1.**
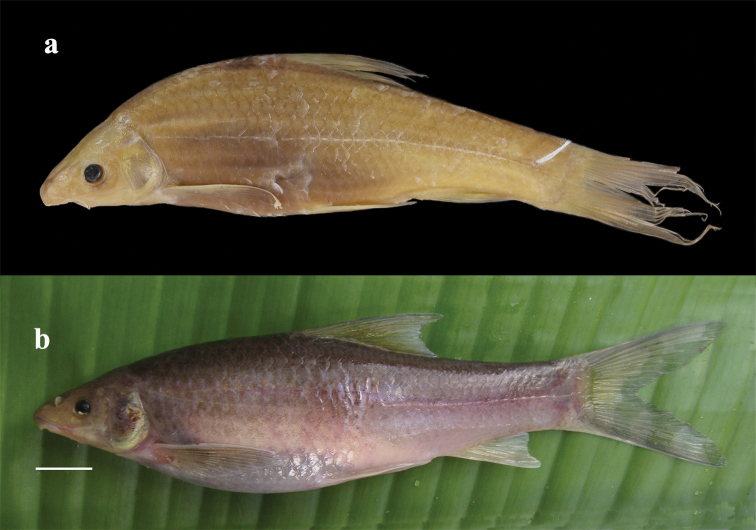
Lateral view of ‘Henicorhynchus’ thaitui sp. nov.: **a** IEBR 105901, holotype, 74.3 mm SL; and **b** IHB 2016105898, paratype, 98.0 mm SL. Both specimens caught in central Vietnam: Son River system in Gianh river drainage: Khe Lanh Cave.

**Figure 2.**
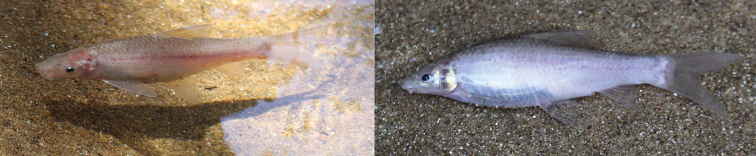
‘Henicorhynchus’ thaitui sp. nov., specimen not preserved, about 100 mm SL, Vietnam: Khe Lanh cave; life coloration.

**Figure 3.**
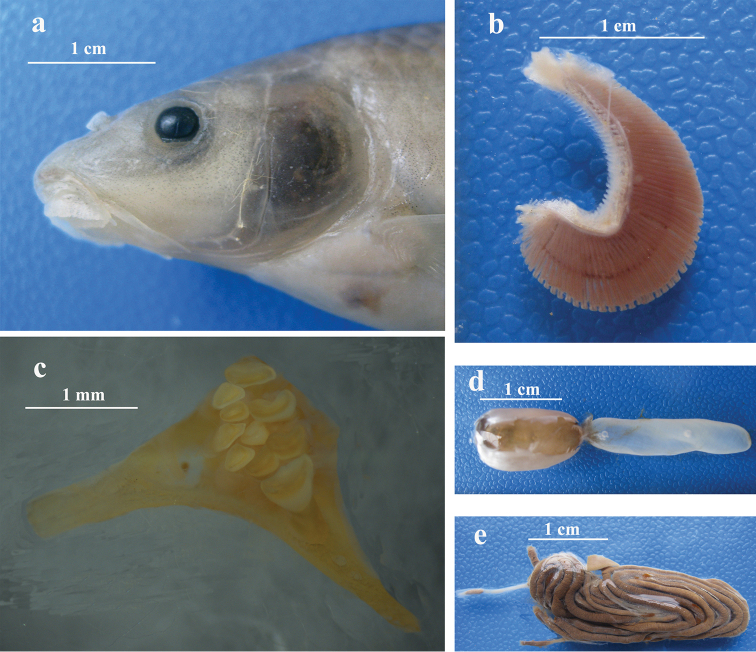
‘Henicorhynchus’ thaitui sp. nov., IHB 2016105898, paratype, 98.0 mm SL: **a** lateral view of head **b** gill rakers on lower arm of first arch **c** pharyngeal teeth **d** air bladder; and **e** intestines.

**Figure 4.**
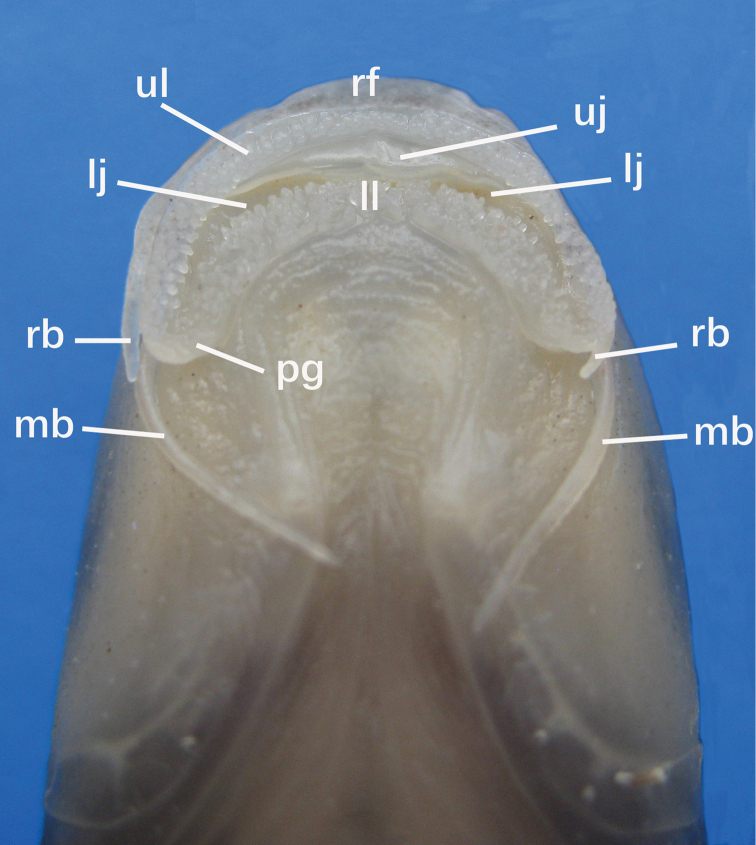
Ventral view of mouthpart structures in ‘Henicorhynchus’ thaitui sp. nov., IHB 2016105898, paratype, 98.0 mm SL. lj, lower jaw; ll, lower lip; mb, maxillary barbel; pg, postlabial groove; rb, rostral barbel; rf, rostral fold; ul, upper lip; uj, upper jaw.

Figures 1 , 2 , 3 , 4

#### Holotype.

IEBR 105901, 74.3 mm SL; Vietnam: Quang Binh Prov.: Phong Nha-Ke Bang National Park: Khe Lanh Cave (in Son River system in the Gianh River basin): 17°25'41"N, 106°18'31"E, altitude 185 m; collectors: A.T. Ho and N.T. Hoang, 12 August 2011 (deposited in IEBR).

#### Paratypes.

IHB 2016105895–9, 5 specimens, 80.9–99.5 mm SL; IEBR 105900–04, 4 specimens, 68.8–75.6 mm SL; VU 5905–08, 4 specimens, 76.8–83.4 mm SL; all other data same as holotype (deposited in IHB and IEBR, VU).

#### Diagnosis.

‘Henicorhynchus’ thaitui sp. nov. can be distinguished from all congeners by having a whitish pink body in life (vs. white or silvery body with a humeral mark or some longitudinal stripes), smaller (vs. larger) eyes (diameter less than vs. greater than maxillary barbel length) and maxillary barbel longer (vs. shorter) than rostral barbel. ‘Henicorhynchus’ thaitui, along with *H. horai* (Bănărescu, 1986) and *H. inornatus* (Roberts, 1997), is further distinct from all other congeners in having 9 (vs. 8) branched dorsal-fin rays. Along with *H. horai*, it differs from all other congeners in the presence of rostral barbels (vs. absent) and 39–41 (vs. 34–36) lateral-line scales. ‘Henicorhynchus’ thaitui differs from *H. horai* and *H. inornatus* in the presence of two (vs. one) pairs of maxillary and rostral barbels (vs. only the maxillary barbel in *H. inornatus* and only the rostral barbel in *H. horai*); from *H. horai* in having fewer vertebrae (34 vs. 38–41), and a laterally compressed body (vs. cylindrical *in H. horai*); and from *H. inornatus* in having 39–40 (vs. 35 in *H. inornatus*) lateral-line pored scales, and in the absence of a humeral mark (vs. present in *H. inornatus*).

#### Description.

Measurements and meristics of the type series are provided in Table 1. See Figs 1, 2 for general appearance, Fig. 3a for lateral and ventral views of the head, and Fig. 4 for morphology of the oromandibular structures. Body elongate and laterally compressed. Dorsal profile of body from tip of snout to dorsal-fin origin slightly convex. Predorsal profile of body convex, without distinctive hump behind head. Postdorsal profile of body slightly concave. Ventral profile of body from tip of snout to anal-fin origin convex; slightly concave from anal-fin origin to origin of ventral procurrent caudal-fin rays.

**Table 1.** Morphometric data and meristic counts for type specimens of ‘Henicorhynchus’ thaitui sp. nov. (n = 14).

	Holotype	Paratypes (N=13)			
		Min	Max	Average	SD
SL	74.3	68.8	99.5	80.9	8.5
**In percent of SL**					
Head length	24.1	20.3	24.1	22.2	0.9
Predorsal length	53.3	47.8	53.3	49.9	1.6
Preanal length	74.2	72.8	77.9	74.6	1.4
Prepelvic length	49.0	49.0	54.4	50.9	1.5
Body depth at dorsal-ﬁn origin	30.0	23.3	30.0	26.9	2.2
Body depth at anus	20.2	16.0	20.2	18.0	1.3
Depth of caudal peduncle	14.2	10.6	14.2	12.7	0.9
Length of caudal peduncle	22.1	13.6	22.1	17.8	2.4
Head depth	11.8	10.9	12.8	11.8	0.6
Head width	11.6	10.4	11.9	11.1	0.6
Snout length	8.8	6.7	9.6	8.4	0.8
Dorsal-fin length	26.3	20.1	29.6	25.7	2.7
Pectoral-fin length	21.9	18.4	25.7	22.6	1.7
Pelvic-fin length	20.6	16.9	23.7	20.8	1.9
Anal-fin length	19.1	14.0	22.7	19.1	2.2
**In percent of HL**					
Head depth	48.9	48.9	57.6	53.1	2.7
Head width	48.4	44.6	54.9	50.0	3.0
Snout length	36.7	32.9	42.5	38.0	3.0
Eye diameter	17.7	10.9	17.7	13.6	1.8
Interorbital width	48.4	44.6	54.9	50.0	3.0
**Counts**					
Lateral line scales	39	39 (11), 40 (2)			
Transverse scale rows	5/1/4	6/1/4(11), 5/1/4 (2)			
Predorsal scales	14	14 (10) –15 (3)			
Cicumpeduncular scales	16	16 (13)			
Dorsal-fin branched rays	9	9 (13)			
Pectoral-fin branched rays	11	11 (13)			
Pelvic-fin branched rays	8	8 (13)			
Anal-fin branched rays	5	5 (13)			
Caudal-fin branched rays	9+8	9+8 (13)			
Gill rakers on lower arm of 1^st^ arch		37–39 (2)			
Vertebrae		34–35 (2)			

Head small, conical, longer than deep, deeper than wide. Eye relatively small, positioned laterodorsally in anterior half of head and considerably behind or above rictus, not visible when head viewed ventrally; diameter less than maxillary-barbel length. Interorbital space slightly convex. Snout slightly pointed in lateral view and broadly rounded in ventral view (Fig. 3a). Nares longitudinal and located closer to orbit than to tip of snout and covered by a flap originating from anterior end. Two pairs of long barbels; maxillary barbel much longer than rostral barbel, extending to posterior margin of orbit or beyond, and rostral barbel extending beyond nostrils but not reaching to anterior margin of orbit. Mouth inferior and slightly arched.

Rostral cap well-developed, overhanging, but covering median part of upper lip base; slightly crenulated, laterally attached to root of maxillary barbel and separated from lower lip. Upper lip well-developed, greatly enlarged, separated from upper jaw, covered with papillae; laterally continuous with lower lip around corners of mouth. Upper jaw bearing a thin, flexible horny sheath on cutting margin. Lower lip anteriorly separated from lower jaw by a deep, transversally arched groove; posteriorly confluent with mental region and covered with papillae. Post-labial groove extended anteromedially, but not meeting its counterpart at midline. Lower jaw bearing a thin horny sheath on cutting margin (Fig. 4)

Dorsal fin with 3 unbranched and 9 branched rays, last one split to base; last unbranched ray flexible, without serrations along posterior border; distal margin strongly concave; origin midway between snout tip and caudal-fin base or anterior to pelvic-fin insertion. Pectoral fin short, with 1 unbranched and 11 branched rays; tip of adpressed fin not reaching pelvic-fin insertion. Pelvic fin falcate, with 1 unbranched and 8 branched rays; inserted halfway from pectoral-fin insertion to anal-fin origin; tip of adpressed fin extending to vent. Anal fin with 3 unbranched and 5 branched rays; distal margin slightly concave; origin equidistant between pelvic-fin insertion and caudal-fin base. Caudal fin with 9/8 principal rays, deeply forked; upper and lower lobes nearly equal in length.

Scales moderately large. Lateral line complete with 39 (12*) or 40 (2) pored scales, extending along mid-lateral body from upper gill-opening extremity to middle of caudal fin. Predorsal scales 14 (10*) or 15 (3). Scales in transverse row before pelvic fin 5 (3) or 6 (12*) above lateral line and 4 (14*) below. Circumpeduncular scales 16 (14*). Gill rakers on left side of first gill arch 37–39 (2) (Fig. 3b). Pharyngeal teeth in 3 rows: 2.4.5–5.4.2 (IHB 2016105898, paratype, Fig. 3c). Air bladder bipartite (Fig. 3d). Intestines long, thin and highly coiled (Fig. 3e). Vertebrae 4+34–35=38–39 (2).

#### Coloration.

In freshly caught-individuals, body white to pinkish or pale pink with all fins translucent (Fig. 2). In captivity, body pale pink but dorsum turning to pale brown hue in adults exposed to light, becoming gray dorsally within several hours when exposed to daylight (Fig. 1b). In alcohol-preserved specimens, body uniformly pale yellow, with all fins light gray, particularly in distal portion (Fig. 1a).

#### Etymology.

The specific name is a noun in the genitive case, honoring Nguyen Thai Tu, ichthyologist from Vinh University, who has contributed considerably to the taxonomy of freshwater fishes in Vietnam.

#### Troglomorphic characters.

‘Henicorhynchus’ thaitui sp. nov. presents a mixture of characters characterizing hypogean and epigean fish species. The absence of pigmentation, reduced eye size, and well-developed barbels are troglomorphic characters observed in ‘H.’ thaitui. The pale pink or white to pinkish body is shared with hypogean fish species. The eyes are smaller than in congeneric epigean species, but not vestigial or absent as is common in hypogean fish species. The barbels are longer and thicker compared with all congeneric epigean species, but in this regard similar to hypogean fish species.

#### Distribution and habitat.

**Figure 5.**
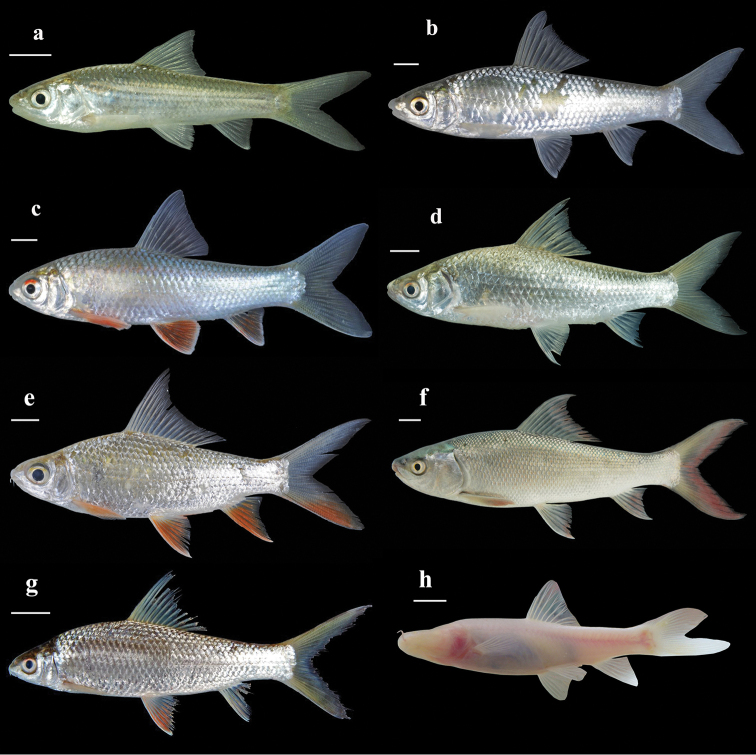
Species most closely related to ‘Henicorhynchus’ thaitui sp. nov.: **a***Henicorhynchus lineatus***b***H. lobatus***c***H. ornatipinnis***d***H. siamensis***e***Cirrhinus jullieni***f***C. microlepis***g***C. molitorella*; and **h***Speolabeo hokhanhi*. Photos **b**, **d**, **e**, **f** and **g** from Vietnam, by D.D. Tran (Can Tho University, Vietnam); **a** and **c** from Laos, by Bounthob Praxaysombath (National University of Laos, Laos) (from Kano et al. 2013); and **h** from Vietnam, by D.T. Nguyen. Scale bars: 1 cm.

**Figure 6.**
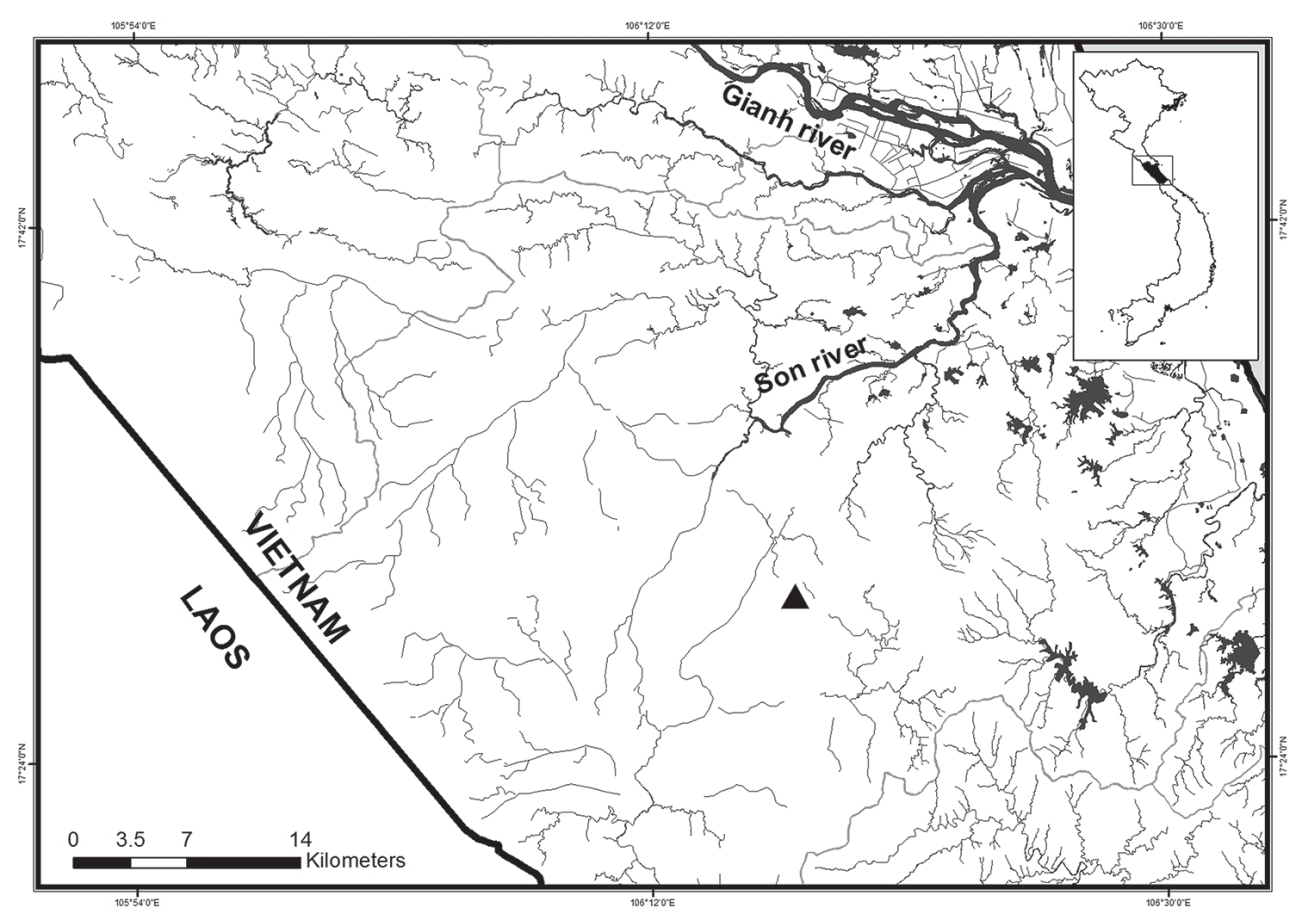
Map showing the distribution of ‘Henicorhynchus’ thaitui sp. nov. (▲).

**Figure 7.**
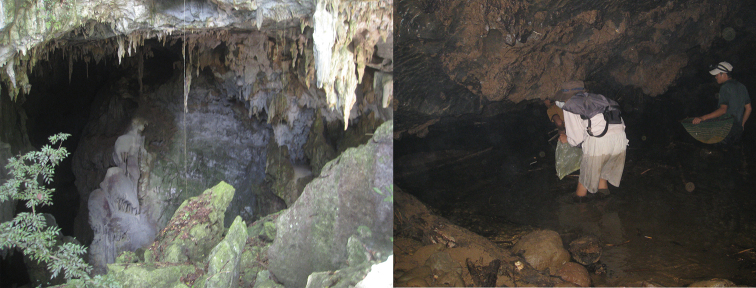
Vietnam: Son River system in Gianh River drainage basin: Khe Lanh Cave: mouth of cave (left) and habitat of ‘Henicorhynchus’ thaitui sp. nov. (right).

‘Henicorhynchus’ thaitui sp. nov. is known only from the Khe Lanh Cave where it inhabits shallow to deep (0.2–0.8 m) cave streams and pools about 800–1000 m from the cave entrance (Figs 5, 6). This cave is located approximately 25 km south of Phong Nha village in the Son Trach commune. It has a length of 1–2 km, completely without light, with a mixed substrate of mud and sands. The type series of ‘H.’ thaitui was collected in August 2011, roughly 1 km from the cave entrance. At least 50 individuals of various sizes were observed in streams and pools, 14 of which were caught by hand net (Fig. 7). The fishes were swimming slowly and haphazardly, rather close to the water surface; they swam deeper when disturbed. A new shrimp species (Do and Nguyen 2014) and the labeonine fish species *Speolabeo hokhanhi* were recently discovered in the Hang Va Cave, 3–5 km away from the Khe Lanh Cave (Nguyen et al. 2018).

**Table 2.** Main diagnostic characters for ‘Henicorhynchus’ thaitui sp. nov. and its congeneric species.

**Characters**	***H. thaitui***	***H. horai***	***H. inornatus***	***H. siamensis***	***H. lineatus***	***H. lobatus***	***H. caudiguttatus***	***H. cryptopogon***	***H. ornatipinnis***	***H. caudimaculatus***
Branched dorsal-fin rays	9	9	9	8	8	8	8	8	8	8
Rostral barbels	present	present	absent	absent	absent	absent	absent	absent	absent	absent
Maxillary barbels	present, long and well developed	absent	absent	present, very tiny	present, tiny	present, tiny	present, tiny	present, tiny	absent	absent
Lateral line scales	39–40	39–41	35	34–36	34–35	32–33	35	34–35	34–35	35–36
Transverse scales rows	5–6/1/4	6/1/4	7/1/5	6/1/4	6/1/5	5/1/4	6/1/5	6/1/5	6/1/4	6/1/5
Vertebrae	34–35	38–41	35–37	33–34	32–33	32	32	32–33	33–35	32
Dark state color distal dorsal-fin edge	absent	absent	absent	present	absent	absent	absent	present	absent	absent
Reddish-orange pectoral fins	absent	absent	absent	absent	absent	absent	absent	absent	present	absent
Longitudinal stripes on body	absent	absent	absent	absent	present	absent	absent	present	absent	absent
Humeral mark color	absent	absent	present	absent	absent	absent	absent	absent	absent	absent
Strongly projected snout	absent	absent	absent	absent	absent	present	absent	absent	absent	present
Dark state color precaudal blotch	absent	absent	absent	absent	absent	absent	present	absent	absent	present
Sources of data	this study	Vidthayanon (2008), Roberts (1997)	Roberts (1997)	Roberts (1997), Rainboth (1996), Kottelat (2001)	Roberts (1997), Kottelat (1998), Kottelat (2001)	Roberts (1997), Kottelat(2001)	Roberts (1997), Rainboth et al. (2012)	Rainboth (1996)	Roberts (1997), Kottelat (2001)	Roberts (1997), Rainboth (1996)

## Discussion

‘Henicorhynchus’ thaitui sp. nov. is referred to the genus *Henicorhynchus* with hesitation. It can be distinguished from all other species of this genus by the troglomorphic characters, and would be the only species of *Henicorhynchus* recorded from a cave biotope.

The karst region of Central Vietnam extends into the central part of Laos from where the labeonine cavefish *Speolabeo musaei* is known. The second species of the genus, also troglobitic, is *S. hokhanhi* (Fig. 5h), recently described by Nguyen et al. (2018) from Central Vietnam. Three main characters typical for *Speolabeo* Kottelat are: 7–8 branched dorsal-fin rays, reduced smooth upper lip and postlabial groove widely interrupted at the isthmus with its counterpart (Nguyen et al. 2018). ‘Henicorhynchus’ thaitui cannot be referred to *Speolabeo*, differing in the presence of 9 branched dorsal-fin rays, a well-developed, greatly enlarged, papillose upper lip and postlabial groove narrowly interrupted at the isthmus with its counterpart.

The Labeonini are characterized by a high degree of morphological modification of their oromandibular structures, variation in which is the basis for the diagnosis of the majority of included genera. The well-developed, greatly enlarged upper lip separated from the upper jaw in ‘H.’ thaitui is shared only with some species of the labeonine genera *Osteochilus* Günther, *Labiobarbus* van Hasselt, *Labeo* Cuvier, *Cirrhinus* and *Henicorhynchus* (Wu et al. 1977; Zhang et al. 2000; Zhang and Chen 2006). It differs from *Osteochilus* (*sensu* Roberts, 1989) in the absence of elongate folds or plicae on the upper lip; and from *Labiobarbus* (*sensu* Rainboth, 1996) in having 9 rather than 18–30 branched rays of the dorsal fin. *Labeo*, as traditionally defined, is widespread in the tropical regions of Asia and Africa (Reid 1985). Its non-monophyly was shown in Yang et al.’s (2012) molecular phylogenetic analysis of the Labeonini; these authors therefore restricted *Labeo* s. str. to the clade sister to ‘Cirrhinus’ microlepis Sauvage, 1878, but upholding this clade may result in multiple generic lineages. In this context, Asian *Labeo* is here confined to species like *Labeo dyocheilus* McClelland, 1839, following Rainboth (1996) and Kottelat (2013). ‘Henicorhynchus’thaitui is not congeneric with Asian species of *Labeo*, lacking a thickened lower lip with a deep postlabial groove narrowly interrupted at the isthmus with its counterpart. It is highly likely that ‘H.’ thaitui is a member of *Henicorhynchus* or *Cirrhinus*, or even of a distinct genus.

‘Henicorhynchus’ thaitui sp. nov. can be distinguished by its troglomorphic characters from all other species of *Henicorhynchus* and closely related genera like *Cirrhinus* and *Gymnostomus*. Except for the two species of *Speolabeo* (Nguyen et al. 2018) and some species of *Garra* Hamilton in the Middle East (Khalaf-Sakerfalke von Jaffa 2009; Hamidan et al. 2014), no species with troglomorphic characters like this are found in the Labeonini.

The generic placement of ‘H.’ thaitui is not straightforward, however. Compared with species of *Henicorhynchus* or closely related genera which have relatively simple oromandibular structures (see Roberts 1997 for details), ‘H.’ thaitui has unique modifications in these structures, including rostral cap pendulous with a slightly crenulated distal margin, laterally attached to the root of the maxillary barbel and discontinuous with the lower lip, and papillated lips (Fig. 4). These characters might justify placing ‘H.’ thaitui in a new genus. Such act will need a more thorough analysis, involving all labeonine genera. For the moment, the recognition of ‘H.’ thaitui as a distinct species, regardless of genus, is justified on account of its restricted distribution and specialized ecology. Anthropogenic activities on both the global and local scales pose a potential threat to survival of all cavefishes, and their identification by a scientific name will facilitate conservation actions.

The taxonomy of *Henicorhynchus* and closely allied genera is poorly understood. Their generic delineation is not based on the morphology of the oromandibular structures, which are widely used for the diagnosis of genera of the Labeonini. The major character used to distinguish among *Henicorhynchus* and closely allied genera (*Cirrhinus* and *Gymnostomus*) is the number of branched dorsal-fin rays; their current generic definition still remains controversial, however. The focus of this controversy is on the generic assignment of species with 9 branched dorsal-fin rays. According to Kottelat (2003), *Cirrhinus* is composed of species with 10–15 branched dorsal-fin rays, and those with 8–9 rays were ascribed to *Gymnostomus*, with which *Henicorhynchus* was synonymized. Referring to Kottelat (2013), six species were assigned to *Cirrhinus*, namely *C. cirrhosis* (Bloch, 1795), *C. jullieni* Sauvage, 1878 (Fig. 5e), *C. microlepis* (Fig. 5f), *C. molitorella* (Valenciennes in Cuvier & Valenciennes, 1844) (Fig. 5g), *C. prosemion* (Fowler, 1934), and *C. rubirostris* Roberts, 1997. Rainboth et al.’s (2012) generic definition of *Henicorhynchus* included the following seven species with 8 branched dorsal-fin rays: *H. lineatus* (Smith, 1945) (Fig. 5a), *H. lobatus* (Fig. 5b), *H. ornatipinnis* (Roberts, 1997) (Fig. 5c), *H. caudimaculatus*, *H. cryptopogon*, *H. siamensis* (Fig. 5d), and *H. caudiguttatus* (Fowler, 1934). These authors ascribed *H. inornatus*, which has 9 branched dorsal-fin rays and which was previously included in *Gymnostomus* by Kottelat (2003), to *Cirrhinus*. Earlier, Roberts (1997) lumped all species with 8–15 branched dorsal-fin rays into *Cirrhinus*. This taxonomic treatment, though, was not widely accepted by subsequent workers.

Taxonomic confusion regarding *Henicorhynchus*, *Gymnostomus*, and *Cirrhinus* was clarified in recent molecular phylogenetic analyses of the Labeonini (Yang et al. 2012; Zheng et al. 2012, 2016). It was demonstrated that sampled species of *Henicorhynchus* nested into an independent lineage, and so did that of *Gymnostomus*; *Cirrhinus* was non-monophyletic, and *C. microlepis* and *C. molitorella* were distantly related to each other or to the rest of the analyzed congeneric species. On the basis of these findings, it can be concluded that *Gymnostomus* [type species: *Cyprinus ariza* Hamilton, 1807, a species designated by Roberts (1997) to *Cirrhinus*] is a valid genus; *Henicorhynchus* possibly includes species with 8 branched dorsal-fin rays; and both *C. molitorella* and *C. microlepis* should be removed from *Cirrhinus*. However, insufficient sampling made it unlikely to reach a decisive conclusion about the generic placement of these three genera. Two species, namely *H. inornatus* and *H. horai*, with 9 branched dorsal-fin rays, were not sampled in Yang et al.’s (2012) analysis, rendering their generic status untested. Fricke et al. (2020) nevertheless referred these two species to *Henicorhynchus*.

## Comparative material

*Henicorhynchus siamensis*: RIA2, uncataloged, 30, 62–184 mm SL, South Vietnam (Lower Mekong basin); CAS 91751, 109 mm, Pak Mun; CAS 91749, 4, 57.4–65.2 mm, Menam Bangpakong at Ban Khao Cha-chan, 19 km South of Sa Kaeo on highway 317.

*H. caudimaculatus*: CTU, uncataloged, 11, 40–80 mm SL, South of Vietnam (Lower Mekong basin); USNM 117769, 27, 45.0–78.5 mm, upper Nan River at Ban Khwang, northern Siam; USNM 119493, 2, 59. 6–60.5 mm, Chao Phraya at Bangsai, central Thailand; CAS 91781, 55.2 mm, Menam Wang, 79 km by road North of Lampang and 6 km E of highway 1035.

*H. cryptopogon*: RIA2, uncataloged, 1, 145 mm SL, Can Tho province.

*H. inornatus*: CAS 91772, 115 mm, Myanmar, Mandalay market; CAS 88903, 106 mm, Pagan market; CAS 91775, 122 mm, Myitkyina market (Irrawaddy basin); CAS 91773, 6: 94.6–116 mm, Taungoo market (Sittang River).

*H. lineatus*: CAS 79169, 5, 64.9–95.2 mm, mouth of Huay Ngao where it flows into Mekong River 1 km South of Ban Chaem Pong (about 30 km South of Chiang Khong); CAS 91766, 9, 54.5–106 mm, Mekong mainstream from Pak Ing to Jom Paeng (about 4–5 km downstream from Pak Ing).

*H. lobatus*: CAS 91769, 8, 56.9–102 mm, Menam Kok at Tha Ton and up to 5 km downstream; CAS 91767, 4, 43.4–50.5 mm, Se Khone at Stung Treng.

*H. horai*: CAS 81548, 24, 95.9–155 mm, Inle Lake.

*H. ornatipinnis*: CAS 91756, 69.7 mm, roadside ditch on highway 24 at km 150, market, 179 km by road E of Nakom Ratchasima, Thailand; CAS 91760, 9, 55.7–90.5 mm, roadside ditch 5–30 km South of Phibun Mangsahan, Thailand.

*Cirrhinus molitorella*: RIA1 H01930101–7, 7, 105–387 mm SL, Red river. IHB 201808027404, 1, Lam river, Nghe An province, Central Vietnam; CAS 79175, 2, 82.8–93.7 mm, Huay Sangkalia, 7 km North of Sangklaburi on road to Chedi Sam Ong.

*C. microlepis*: RIA1 H01930201–3, 3, 65–148 mm SL, south of Vietnam; USNM 104935, 2, 122–146 mm, Chao Phraya at Nontaburi; CAS 79173, 2: 107–113 mm, rapids in mainstream of Mekong River about 12 km South of That Phanom.

*C. mrigala* (Hamilton, 1822): RIA1 H01930301–5, 5, 150–258 mm SL, aquaculture. IHB 201808018201–5, 5, Lam river, Nghe An province, Central Vietnam.

*C. prosemion*: CTU, uncataloged, 11, 71–95 mm SL, Can Tho province.

*C. jullieni*: CAS 91748, 104 mm, Sawankhalok market (Menam Yom), 36 km North of Sukhothai; CAS 91610, 13: 94.7–104 mm, Phnom Penh market.

*Speolabeo hokhanhi*: IHB 2016092883, 76.4 mm, Hang Va cave, Phong Nha-Ke Bang National Park; IEBR 2884–5, 50.7–54.4 mm, IHB 206092886–8, 61.8–69.0 mm.

Data for *Cirrhinus reba* (Hamilton, 1822) from Vidthayanon et al. (2005) and Vidthayanon (2008); for *C. cirrhosis*, *C. rubirostris*, *Gymnostomus fulungee* (Sykes, 1839), and *G. ariza* from Roberts (1997).

## Supplementary Material

XML Treatment for ‘Henicorhynchus’ thaitui
